# Association of cluster determinant 36, scavenger receptor class B type 1, and major facilitator superfamily domain containing the 2a genetic polymorphism with serum lipid profile in aging population with type 2 diabetes mellitus

**DOI:** 10.3389/fnut.2022.981200

**Published:** 2022-09-14

**Authors:** Xixiang Wang, Xiaojun Ma, Jingjing Xu, Yujie Guo, Shaobo Zhou, Huiyan Yu, Linhong Yuan

**Affiliations:** ^1^School of Public Health, Capital Medical University, Beijing, China; ^2^School of Science, Faculty of Engineering and Science, University of Greenwich, Chatham, United Kingdom

**Keywords:** serum lipid profile, type 2 diabetes mellitus, lipid metabolism-related gene, genetic polymorphism, the elderly

## Abstract

**Background:**

Lipid metabolism disorder commonly happens in subjects with Type 2 diabetes mellitus (T2DM) which may be linked to genetic variants of lipid metabolism-related genes. However, few studies have explored the relationship between lipid metabolism-related gene polymorphism and serum lipid profile in aging subjects with T2DM. The present study was designed to explore the impact of genetic polymorphism of cluster determinant 36 (*CD36*) (rs1049673, rs1054516, rs2151916), scavenger receptor class B type 1 (*SCARB1*) (rs5888), and major facilitator superfamily domain containing the 2a (*MFSD2A*) (rs12083239, rs4233508, rs12072037) on the relationship between circulating lipids in aging subjects with T2DM.

**Methods:**

205 T2DM patients and 205 age and gender matched control subjects were recruited. Information on demographic characteristics was collected by using a self-administered questionnaire. Fasting venous blood samples were taken for lipid-related gene genotyping and serum lipid profile measurement. The Chi-square test was used to compare percentage differences and to calculate *P*-value for Hardy-Weinberg equilibrium. Logistic regression and multiple linear regression were used to explore the risk or correlation between variables, and general linear model (GLM) was used to compare the means of serum lipids between the groups.

**Results:**

In T2DM group, *CD36* rs1054516 and *MFSD2A* rs12072037 were correlated with serum TC level. In control group, *CD36* rs1049673 was correlated with serum HDL-C level. Meanwhile, T2DM subjects with *MFSD2A* rs12083239 (CG), *MFSD2A* rs4233508 (TT), and *MFSD2A* rs12072037 (AA) had higher TG level than control subjects. T2DM subjects with *CD36* rs1049673 (CG, GG), *CD36* rs1054516 (CT), *CD36* rs2151916 (TT, CT), *SCARB1* rs5888 (GG), *MFSD2A* rs12083239 (GG, CG), *MFSD2A* rs4233508 (TT), and *MFSD2A* rs12072037 (CA, AA) had lower HDL-C level than control subjects. T2DM subjects with *MFSD2A* rs12072037 (AA) had lower LDL-C level than control subjects. In dominant model, major genotype (GG) of *SCARB1* gene was associated with the risk of T2DM (OR = 0.636, *P* = 0.032).

**Conclusion:**

The genetic polymorphism of *CD36* (rs1049673, rs1054516, rs2151916), *SCARB1* (rs5888), and *MFSD2A* (rs12083239, rs4233508, rs12072037) were associated with serum lipids in T2DM subjects. The *SCARB1* rs5888 major genotype (GG) was a protective factor for T2DM. Large scale cohort study is required to determine the relationship between lipid metabolism-related gene polymorphism, serum lipid profile and T2DM in aging subjects.

## Introduction

Type 2 diabetes mellitus (T2DM) is a chronic metabolic disease that commonly happens in the middle-aged and aged population. The typical clinical characteristics of T2DM are the disorder of glucose and lipid metabolism, manifested by increased plasma the level of glucose and insulin and compensatory insulin secretion insufficiency (1, 2). Moreover, the lipid metabolism disorder can lead to increased circulating free fatty acids (FFA) level, and adipose ectopia deposit in non-adipose tissues, which partially damage the function of islet β cells (3). Hence, the intervention on the FFA transporter system is one of the therapeutic targets to improve dyslipidemia and slow down the process of T2DM.

Lipid metabolism is involved with various FFA transporters. Scavenger Receptor (SR-B), as a member of the FFA transporter system, was reported directly to participate in lipid metabolism through mediating lipoprotein uptake. SR-B includes the subtypes of fatty acid translocase cluster determinant 36 (CD36) and scavenger receptor class B type 1 (SCARB1). The *CD36* gene is highly polymorphic and it locates on the q11.2 region of human chromosome 7, has 15 exons (4). CD36 is extensively expressed in platelets, adipocytes, cardiomyocytes, and macrophages. CD36 is a complex multifunctional protein that acts as a scavenging receptor for oxidized low-density lipoprotein (ox-LDL) and apoptotic cells, as well as a fatty acid transporter (5). Another subtype, *SCARB1* gene locates on chromosome 12 and consists of 13 exons and 12 introns (6). SCARB1 is a polyligand membrane receptor protein, involved in cholesterol reverse transport and regulation of HDL metabolism (7). The major facilitator superfamily domain containing the 2a (*MFSD2A*) gene locates on chromosome 1, 14.3 kb in length and consists of 14 exons and 13 introns. MFSD2A is extensively expressed in brain, liver and brown fat tissues. MFSD2A mainly participants in the cellular uptake of n-3 polyunsaturated fatty acids (n-3 PUFAs) (8, 9).

Genetic susceptibility plays a vital role in the pathology of T2DM, and there are increasing evidence indicated that the genetic architecture of T2DM is polygenic (10–12). Several studies have pointed out that single nucleotide polymorphisms (SNPs) of specific genes, such as *mir-143/145* (13), *IGFBP2* (14), *NR1H2* (15), *SLC30A8* (16), *miR146a* (17), and *ADRB-1* (18), are associated with susceptibility to T2DM. These data highly indicate the importance of exploring genetic polymorphism’s role in the risk of T2DM. Results of meta-analysis had shown that genetic variants in low density lipoprotein cholesterol-lowering gene were associated with an increased risk of T2DM (19), suggesting that the SNPs of lipid metabolism-related genes is possibly associated with T2DM. Previous study had indicated an association between the *CD36* gene polymorphism with the risk of T2DM (20). Studies found that the elevation of CD36 protein increases cellular FFA uptake and correlates with insulin resistance in diabetes (21, 22), demonstrating the potential role of CD36 in affecting glucose and lipid metabolism in T2DM. Besides, the correlation between the *SCARB1* gene polymorphism and insulin sensitivity was also reported by previous study (23), which suggests the involvement of *SCARB1* in the metabolism disorder of glucose and lipid in T2DM. Data from animal experiments also showed that *MFSD2A* knockout mice demonstrate reduced triglyceride (TG) level in serum, liver, and brown adipose tissue (24), suggesting the important role of *MFSD2A* in affecting lipid metabolism.

CD36 and SCARB1 were found to mediate the uptake of lipoproteins, and MFSD2A was reported to involve in DHA transport. All these molecules play pivotal role in lipids metabolism, as well as correlate with susceptibility to T2DM or glucagon signaling. Given these facts, we therefore speculate that the genetic polymorphism of these lipid metabolism-related genes might modify the correlation of serum lipids with T2DM, especially in the aging population prone to lipid metabolism disorder. Therefore, in the current study, an aging population-based case-control study was conducted to explore the correlation of serum lipids with T2DM in the subjects with different *CD36*, *SCARB1* and *MFSD2A* genotypes. Our study will provide basic evidence for setting precision lipid management strategies in the diabetic population based on their genetic background.

## Materials and methods

### Study population

205 T2DM patients and 205 age (±2 years old) and gender matched control subjects were recruited in Nanyuan Community (Beijing, China) and Wulituo Community (Beijing, China). There were 73 men and 132 women in each group. Diabetes patients were determined according to guidelines for the prevention and control of type 2 diabetes in China (2017 Edition, Chinese Diabetes Society), with typical symptoms and fasting blood glucose ≥ 7.0 mmol/L or postprandial blood glucose ≥ 11.1 mmol/L. The study protocol was approved by the Committee on Medical Ethics of Capital Medical University (No. 2012SY23). The study procedures followed the ethical standards of the Helsinki Declaration of 1975. Informed consent was signed by all participants.

### Demographic characteristics and anthropometric measures

Participants underwent a general information survey, medical history survey and physical examination. Information on demographic characteristics (age, gender), lifestyle factors [smoking (yes or no), alcohol drinking (yes or no) and physical activity (never, 1–3 times/week, 4–5 times/week, everyday)], and medical history of chronic disease [hyperlipidemia (yes or no), hypertension (yes or no), coronary heart disease (yes or no), stroke (yes or no) and atherosclerosis (yes or no)] were collected by using a self-administered questionnaire. The nurses of the community medical service center documented anthropometric measures (height and weight). Body mass index (BMI) was calculated as weight (kg)/height (m^2^).

### Biochemical measurements

The fasting venous blood (5 mL) was collected from all the participants in the morning. After centrifuging at 1,500 g for 15 min, the plasma was separated and used for lipid parameter measurement. ILAB600 clinical chemistry analyzer (Instrumentation Laboratory, Lexington, WI, United States) was applied for glucose (GLU), total cholesterol (TC) and triglyceride (TG) measurement. High-density lipoprotein cholesterol (HDL-C) was determined by a commercially available assay from the Instrumentation Laboratory (Lexington, WI, United States), and low-density lipoprotein cholesterol (LDL-C) was calculated according to the Friedewald formula (25). All samples for each participant were analyzed within a single batch, and the inter-assay coefficients of variation (CV) were less than 5%.

### Genotyping

The Wizard genomic DNA purification kit (Promega, Madison, WI, United States) was used to extract DNA from the whole blood sample. The genetic polymorphism of *CD36* (rs1049673, rs1054516, rs2151916), *SCARB1* (rs5888), *MFSD2A* (rs12083239, rs4233508, rs12072037) was detected by SNPscan genotyping assay according to the method descripted by previous study (26).

### Statistical analysis

All data were statistically analyzed by SPSS 26.0 software (Chicago, IL, United States). Graphs were prepared by GraphPad Prism 8.0 (GraphPad Software). Continuous variables were represented as mean ± standard deviation (*SD*). Chi-square test was used to compare the differences in percentages and assess Hardy-Weinberg equilibrium. The allele and genotype frequency were calculated from the observed genotypic counts. The effect of different SNPs on the risk of T2DM in different gene models by using logistic regression. Multiple linear regression was applied to ascertain the correlation between alleles or genotypes and serum lipids. General linear model (GLM) was used to compare the means of the detected parameters between the groups. Potential confounding factors, including sex, age, BMI, hyperlipidemia, hypertension, coronary heart disease, atherosclerosis, smoking, alcohol drinking and physical activity, were adjusted during data analysis. The power was analyzed by using Gpower 3.1 software. P < 0.05 was considered as significant.

## Results

### Demographic characteristics and plasma lipid levels of the subjects

The demographic characteristics and plasma lipid levels of the participants are shown in Table 1. The age of the subjects ranged from 58 to 83 years, with an average age of 66.07 ± 5.44 years. There was no significant difference in age, gender (36% of participates are male) and BMI between T2DM and control groups (*P* > 0.05). The number of subjects with hyperlipidemia, hypertension, coronary heart disease and atherosclerosis in the T2DM group was significantly higher than that in the control group (*P* < 0.05). There was no significant difference in lifestyle (including smoking, alcohol drinking, and physical activity) and stroke between groups. Compared with the control subjects, the T2DM subjects had higher plasma GLU and lower HDL-C levels (*P* < 0.001). There was no significant difference in TC, TG and LDL-C levels between groups.

**TABLE 1:** Demographic characteristics and plasma lipids in T2DM and control subjects.

Demographic characteristics	T2DM (*n* = 205)	Control (*n* = 205)	*P-value*
Age (years)	66.08 ± 5.51	66.06 ± 5.38	0.964
Gender, *n* (%)			1.000
Male	73 (35.6)	73 (35.6)	
Female	132 (64.4)	132 (64.4)	
BMI (kg/m^2^)	25.51 ± 3.43	25.22 ± 3.41	0.528
Smoking, *n* (%)			0.898
Yes	38 (18.5)	37 (18.0)	
No	167 (81.5)	168 (82.0)	
Alcohol drinking, *n* (%)			0.273
Yes	53 (25.9)	63 (30.7)	
No	152 (74.1)	142 (69.3)	
Physical activity, *n* (%)			0.072
Never	21 (10.2)	14 (6.8)	
1–3 d/w	18 (8.8)	31 (15.1)	
4–6 d/w	18 (8.8)	26 (12.7)	
Everyday	148 (72.2)	134 (65.4)	
Hyperlipidemia, *n* (%)			0.000
Yes	98 (47.8)	57 (27.8)	
No	107 (52.2)	148 (72.2)	
Hypertension, *n* (%)			0.000
Yes	132 (64.4)	96 (46.8)	
No	73 (35.6)	109 (53.2)	
Coronary heart disease, *n* (%)			0.018
Yes	56 (27.3)	36 (17.6)	
No	149 (72.7)	169 (82.4)	
Stroke, *n* (%)			0.283
Yes	14 (6.8)	9 (4.4)	
No	191 (93.2)	196 (95.6)	
Atherosclerosis, *n* (%)			0.044
Yes	35 (17.1)	21 (10.2)	
No	170 (82.9)	184 (89.8)	
GLU (mmol/L)	7.27 ± 2.39	5.21 ± 1.16	0.000
TC (mmol/L)	4.86 ± 1.10	4.96 ± 0.97	0.554
TG (mmol/L)	1.93 ± 1.26	1.71 ± 1.02	0.184
HDL-C (mmol/L)	1.27 ± 0.26	1.38 ± 0.31	0.000
LDL-C (mmol/L)	3.12 ± 0.98	3.16 ± 0.91	0.827

Data were expressed as means ± SD or n (%). Demographic characteristics, including age and BMI, were compared by using t-tests. Gender, lifestyle and medical history of chronic disease were compared by using Chi-square tests. GLU, TC, TG, HDL-C and LDL-C levels were compared by general linear model (GLM). Confounding factors, including sex, age, BMI, hyperlipidemia, hypertension, coronary heart disease, atherosclerosis, smoking, alcohol drinking and physical activity, were adjusted during data analysis. P < 0.05 was considered as significant. T2DM, diabetes mellitus type 2; BMI, body mass index; GLU, glucose; TC, total cholesterol; TG, triglyceride; HDL-C, high-density lipoprotein cholesterol; LDL-C, low-density lipoprotein cholesterol.

### Correlation of plasma lipids with the risk of type 2 diabetes mellitus

**FIGURE 1:**
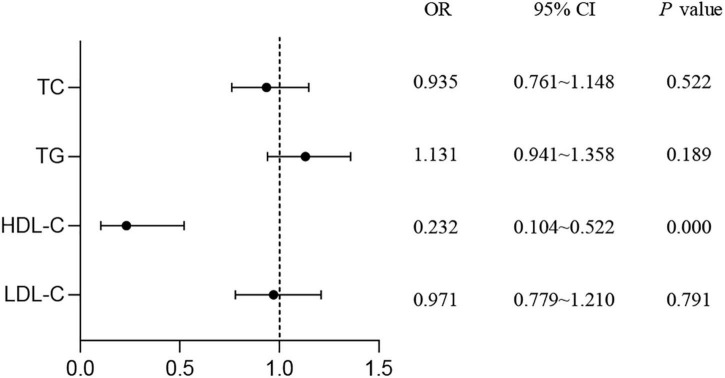
Study on the correlation of serum lipids with the risk of T2DM. Logistic regression was used to detect the risk of lipid levels for T2DM. *P* < 0.05 was considered as significant. TC, total cholesterol; TG, triglyceride; HDL-C, high-density lipoprotein cholesterol; LDL-C, low-density lipoprotein cholesterol; T2DM, type 2 diabetes mellitus; OR, odds ratio; CI, confidence interval.

As shown in Figure 1, the results of logistic regression analysis showed that HDL-C was a protective factor for T2DM (OR = 0.232, 95% CI: 0.104–0.522, *P* < 0.001). No association was found between T2DM risk and TC, TG, or LDL-C levels.

### The allele and genotype distribution of candidate genes in the type 2 diabetes mellitus and control groups

The allele and genotype frequency of the lipid metabolism-related genes in the T2DM and control groups are shown in Table 2. The genotype distribution of the 7 SNPs fitted the Hardy-Weinberg equilibrium (*P* > 0.05). No significant statistical difference was found in the allele frequency of *SCARB1* rs5888 between T2DM and the control groups. However, the difference of *SCARB1* rs5888 genotype frequency was detected between T2DM and control groups. Compared to the control group, a higher percentage of subjects with heterozygote (G/A) but a lower percentage of subjects with major (G/G) or minor (A/A) genotype were found in the T2DM group (*P* < 0.05). There was no significant difference in the allele and genotype frequency of the other SNPs between T2DM and control subjects (*P* > 0.05).

**TABLE 2:** The allele and genotype distribution of candidate genes in the T2DM and control subjects.

SNPs	Groups	Allele [*n* (%)]		*P*-value	Genotype [n (%)]			*P*-value	HWE
						
		Major	Minor		Major type	Heterozygote	Minor type		
***CD36***									
**rs1049673**		C	G	0.530	CC	GC	GG	0.740	0.237
T2DM		214 (52.2)	196 (47.8)		60 (29.3)	94 (45.9)	51 (24.9)		
Control		205 (50.0)	205 (50.0)		53 (25.9)	99 (48.3)	53 (25.9)		
***rs1054516***		T	C	0.889	TT	CT	CC	0.969	0.428
T2DM		215 (52.4)	195 (47.6)		59 (28.8)	97 (47.3)	49 (23.9)		
Control		217 (52.9)	193 (47.1)		59 (28.8)	99 (48.3)	47 (22.9)		
**rs2151916**		T	C	0.688	TT	CT	CC	0.728	0.602
T2DM		304 (74.1)	106 (25.9)		115 (56.1)	74 (36.1)	16 (7.8)		
Control		309 (75.4)	101 (24.6)		116 (56.6)	77 (37.6)	12 (5.9)		
***SCARB1***									
***rs5888***		G	A	0.124	GG	GA	AA	0.011	0.291
T2DM		299 (72.9)	111 (27.1)		106 (51.7)	87 (42.4)	12 (5.9)		
Control		318 (77.6)	92 (22.4)		130 (63.4)	58 (28.3)	17 (8.3)		
***MFSD2A***									
***rs12083239***		G	C	0.443	GG	CG	CC	0.719	0.768
T2DM		327 (79.8)	83 (20.2)		130 (63.4)	67 (32.7)	8 (3.9)		
Control		318 (77.6)	92 (22.4)		122 (59.5)	74 (36.1)	9 (4.4)		
***rs4233508***		C	T	0.530	CC	CT	TT	0.647	0.554
T2DM		202 (49.3)	208 (50.7)		53 (25.9)	96 (46.8)	56 (27.3)		
Control		211 (51.5)	199 (48.5)		54 (26.3)	103 (50.2)	48 (23.4)		
***rs12072037***		C	A	0.722	CC	CA	AA	0.916	0.307
T2DM		240 (58.5)	170 (41.5)		67 (32.7)	106 (51.7)	32 (15.6)		
Control		245 (59.8)	165 (40.2)		71 (34.6)	103 (50.2)	31 (15.1)		

Data were expressed as n (%). Allele frequency, genotype frequency, and HWE were compared by using Chi-square tests. P < 0.05 was considered as significant. SNP, single nucleotide polymorphism; CD36, fatty acid translocase cluster determinant 36; SCARB1, scavenger receptor class B type 1; MFSD2A, major facilitator superfamily domain containing 2a; HWE, Hardy-Weinberg equilibrium; T2DM, diabetes mellitus type 2.

### Association of lipid metabolism-related gene variants with the risk of type 2 diabetes mellitus

As shown in Table 3, the results of logistic regression analysis found that major allele homozygotes (GG) of *SCARB1* rs5888 negatively correlated with the risk of T2DM in aging subjects with the OR value of 0.636 (95% CI: 0.421–0.962, *P* = 0.032) in dominant model. There was no statistical association between other SNPs and the risk of T2DM in aging subjects.

**TABLE 3:** Association of SNPs of candidate genes and odds ratio to T2DM risk.

Genes	SNPs	Additive model		Dominant model		Recessive model	
				
		*P*-value	*OR* 95% CI	*P*-value	OR 95% CI	*P*-value	*OR* 95% CI
***CD36***							
rs1049673 (C > G)		0.929	0.987 0.746∼1.307	0.532	1.156 0.734∼1.822	0.622	0.899 0.557∼1.419
rs1054516 (T > C)		0.983	1.003 0.753∼1.336	0.873	0.964 0.614∼1.514	0.892	1.034 0.635∼1.684
rs2151916 (T > C)		0.637	1.083 0.778∼1.506	0.623	0.902 0.598∼1.361	0.845	0.922 0.407∼2.087
***SCARB1***							
rs5888 (G > A)		0.158	1.264 0.913∼1.749	0.032	0.636 0.421∼0.962	0.492	1.324 0.595∼2.946
***MFSD2A***							
rs12083239 (G > C)		0.284	0.822 0.574∼1.177	0.280	1.263 0.826∼1.931	0.648	1.271 0.454∼3.553
rs4233508 (C > T)		0.653	1.067 0.804∼1.417	0.990	0.997 0.627∼1.585	0.468	0.841 0.528∼1.341
rs12072037 (C > A)		0.899	1.020 0.756∼1.375	0.832	0.955 0.621∼1.467	0.969	1.011 0.578∼1.769

SNPs were expressed as gene names (major allele > minor allele). Additive model: heterozygotes and minor allele homozygotes were weighed 1 and 2, respectively, to major allele homozygotes. Dominant model: major allele homozygotes vs. heterozygotes plus minor allele homozygotes. Recessive model: major allele homozygotes plus heterozygotes vs. minor allele homozygotes. The effect of different SNPs on the risk of T2DM in different gene models by using logistic regression. Adjust for sex, age, BMI, hyperlipidemia, hypertension, coronary heart disease, atherosclerosis, smoking, alcohol drinking and physical activity. P < 0.05 was considered as significant. SNP, single nucleotide polymorphism; CD36, fatty acid translocase cluster determinant 36; SCARB1, scavenger receptor class B type 1; MFSD2A, major facilitator superfamily domain containing 2a; OR, odds ratio; CI, confidence interval.

### Correlation of alleles or genotypes with plasma lipids

The result of multiple linear regression analysis between alleles or genotypes and plasma lipids is shown in Table 4. Plasma lipid levels were associated with allele or genotype of both *CD36* and *MFSD2A* in T2DM subjects and control subjects (*P* < 0.05). The allele and genotype of *CD36* rs1054516 were associated with TC in the T2DM population (*P* < 0.05). In T2DM subjects, plasma TC level also was *MFSD2A* rs12072037 genotype related (*P* < 0.05); and LDL-C level was *CD36* rs1054516 genotype related (*P* < 0.05). The allele and genotype of *CD36* rs1049673 were associated with HDL-C in the control subjects (*P* < 0.05).

**TABLE 4:** Correlation of alleles or genotypes and plasma lipids in the different groups.

Plasma lipids	Groups	Allele/genotype	Unstandardized coefficient	Std. error	Standardized coefficient	*t*	*P*-value
TC	T2DM	*CD36* rs1054516 allele	0.426	0.163	0.176	2.606	0.010
HDL-C	Control	*CD36* rs1049673 allele	0.104	0.046	0.147	2.255	0.025
TC	T2DM	*CD36* rs1054516 genotype	0.302	0.102	0.200	2.964	0.003
TC	T2DM	*MFSD2A* rs12072037 genotype	–0.251	0.109	–0.155	–2.311	0.022
HDL-C	Control	*CD36* rs1049673 genotype	0.071	0.028	0.164	2.500	0.013
LDL-C	T2DM	*CD36* rs1054516 genotype	0.191	0.094	0.142	2.035	0.043

Multiple linear regression was used to ascertain the correlation between alleles (the minor allele non-carrier = 1, the minor allele carrier = 2) or genotypes (CD36 rs1049673: CC = 1, GC = 2, GG = 3; CD36 rs1054516: TT = 1, CT = 2, CC = 3; CD36 rs2151916: TT = 1, CT = 2, CC = 3; SCARB1 rs5888: GG = 1, GA = 2, AA = 3; MFSD2A rs12083239: GG = 1, CG = 2, CC = 3; MFSD2A rs4233508: CC = 1, CT = 2, TT = 3; MFSD2A rs12072037: CC = 1, CA = 2, AA = 3) and plasma lipids in T2DM subjects, and control subjects; respectively. Sex, age, BMI, hyperlipidemia, hypertension, coronary heart disease, atherosclerosis, smoking, alcohol drinking and physical activity have been adjusted for the statistical analysis. P < 0.05 was considered as significant. TC, total cholesterol; HDL-C, high-density lipoprotein cholesterol; LDL-C, low-density lipoprotein cholesterol; T2DM, diabetes mellitus type 2; CD36, Fatty acid translocase cluster determinant 36; MFSD2A, major facilitator superfamily domain containing 2a.

### Serum lipids according to genotype in type 2 diabetes mellitus and control subjects

**FIGURE 2:**
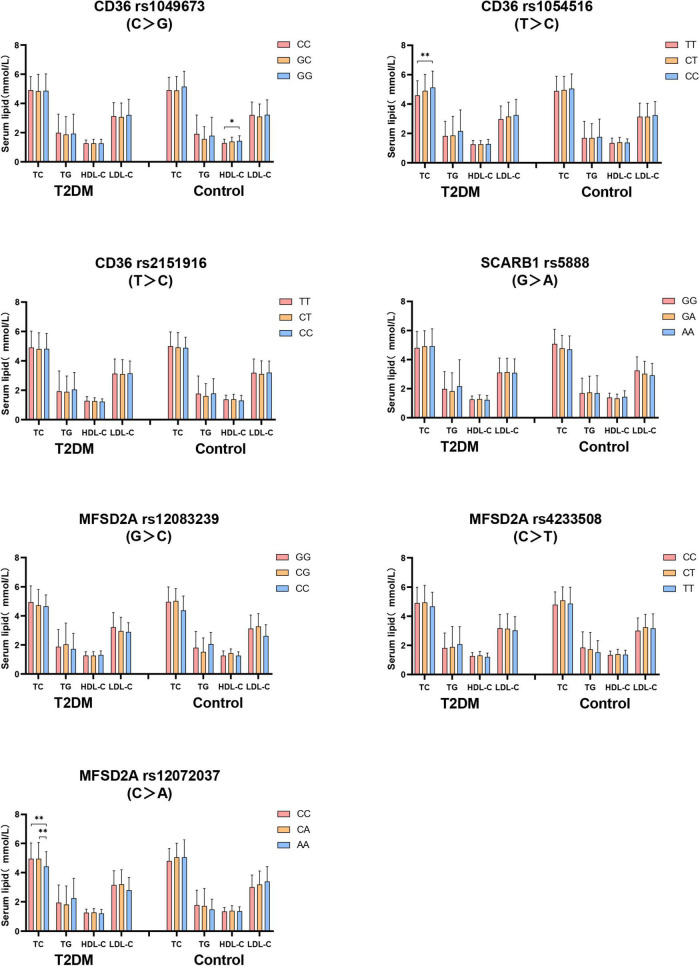
The difference of serum lipid between T2DM and control groups according to different genotypes. General linear model (GLM) was used to compare the means of the detected serum lipids between the groups. *P* < 0.05 was considered as significant (*P < 0.05; **P ≤ 0.01). Sex, age, BMI, hyperlipidemia, hypertension, coronary heart disease, atherosclerosis, smoking, alcohol drinking, and physical activity have been adjusted for the statistical analysis. TC, total cholesterol; TG, triglyceride; HDL-C, high-density lipoprotein cholesterol; LDL-C, low-density lipoprotein cholesterol, T2DM, type 2 diabetes mellitus; CD36, Fatty acid translocase cluster determinant 36; SCARB1, scavenger receptor class B type 1; MFSD2A, major facilitator superfamily domain containing 2a.

Firstly, the difference of serum lipid was compared between T2DM and control groups according to different genotypes. As shown in Figure 2, that the relationship between serum TC level with both *CD36* rs1054516 and *MFSD2A* rs12072037 was found in T2DM subjects. Subjects with *CD36* rs1054516 minor genotype (CC) had higher TC level than subjects with major genotype (TT) (*P* < 0.05); and the subjects with *MFSD2A* rs12072037 minor genotype (AA) had lower TC level than the major allele (CC, CA) carrier (*P* < 0.05). In control group, subjects with *CD36* rs1049673 minor genotype (GG) had higher HDL-C level than major genotype (CC) (*P* < 0.05). There was no difference of serum TC, TG, HDL-C and LDL-C levels between groups according to different genotypes (*P* > 0.05).

**FIGURE 3:**
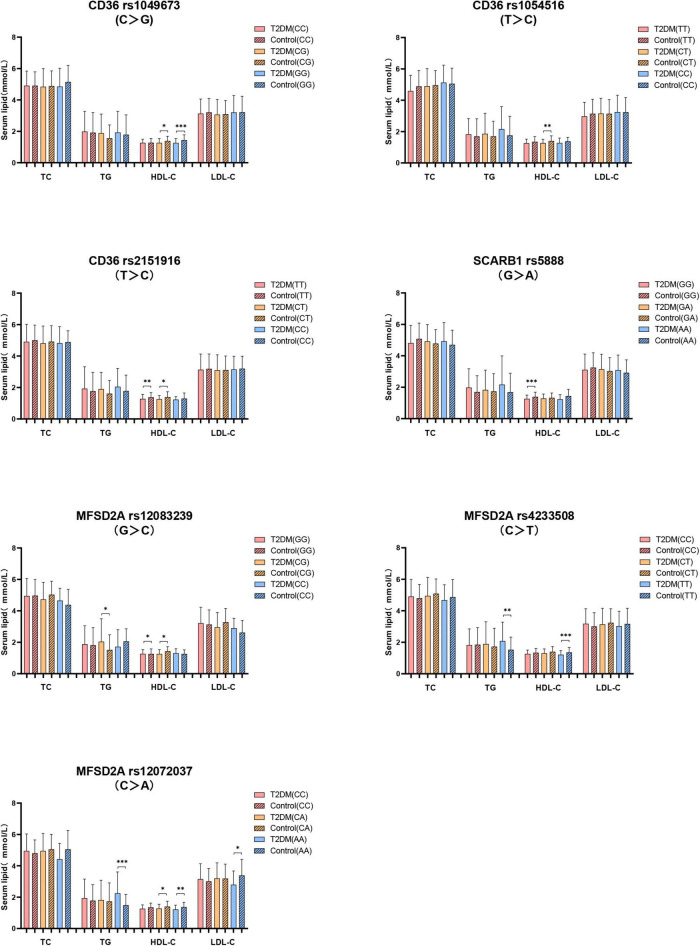
The difference of serum lipids between subjects with different genotypes according to whether they were T2DM patients. General linear model (GLM) was used to compare the means of the detected serum lipids between the groups. *P* < 0.05 was considered as significant (*P < 0.05; **P ≤ 0.01; ***P ≤ 0.001). Sex, age, BMI, hyperlipidemia, hypertension, coronary heart disease, atherosclerosis, smoking, alcohol drinking, and physical activity have been adjusted for the statistical analysis. TC, total cholesterol; TG, triglyceride; HDL-C, high-density lipoprotein cholesterol; LDL-C, low-density lipoprotein cholesterol, T2DM, type 2 diabetes mellitus; CD36, Fatty acid translocase cluster determinant 36; SCARB1, scavenger receptor class B type 1; MFSD2A, major facilitator superfamily domain containing 2a.

Secondly, the serum lipids between subjects with different genotypes were compared according to whether they were T2DM patients. As shown in Figure 3, no difference of serum TC level in subjects with different genotypes between T2DM and control groups. T2DM subjects with *MFSD2A* rs12083239 (CG), *MFSD2A* rs4233508 (TT) and *MFSD2A* rs12072037 (AA) displayed higher TG level than the control subjects (*P* < 0.05). Serum HDL-C level was associated with the genetic polymorphism of *CD36*, *SCARB1* and *MFSD2A*. T2DM patients with *CD36* rs1049673 (CG, GG), *CD36* rs1054516 (CT), *CD36* rs2151916 (TT, CT), *SCARB1* rs5888 (GG), *MFSD2A* rs12083239 (GG, CG), *MFSD2A* rs4233508 (TT) and *MFSD2A* rs12072037 (CA, AA) genotypes had lower HDL-C level than the control ones (*P* < 0.05). And the T2DM subjects with *MFSD2A* rs12072037 (AA) genotype showed lower LDL-C level than the control subjects (*P* < 0.05).

## Discussion

Studies found that elevation of insulin during insulin resistance affects the lipid metabolism (27, 28), and resulting in the disturbed plasma lipid level (29, 30). Jaiswal et al. proposed that T2DM patients mostly have the characteristic pattern of diabetic dyslipidemia, which was demonstrated by higher TG level, lower HDL-C level and the predominance of small dense LDL particles (31). Consistent with these reports, in our study, we found that the prevalence of hyperlipidemia (47.8% vs. 27.8%) and atherosclerosis (17.1% vs. 10.2%), as well as lower HDL-C level in the T2DM patients and they were significantly higher than that in the control subjects, indicating that the T2DM patients were associated with increased risk to have lipid metabolic disorder-related chronic diseases.

The association between insulin resistance and low circulating HDL-C level was found in T2DM patients (31–33). Elevating HDL-C level could reduce blood glucose level through various mechanisms, such as affecting pancreatic beta cells secret insulin, promoting insulin-independent glucose uptake, and enhancing tissue sensitivity to insulin (34, 35). The Prevention of Renal and Vascular End-Stage Disease (PREVEND) study reported that higher circulating HDL-C level predicted decreased the risk for T2DM with OR value was 0.55 (95% CI: 0.47–0.64) (36), suggesting HDL-C was a potential protective factor for T2DM. Consistently, in our study, we found that T2DM patients displayed lower plasma HDL-C level than the control ones, and the results of regression analysis also indicated a significant protective role of HDL-C in decreasing the risk for T2DM (OR = 0.232, 95% CI: 0.104–0.522). The study reported a “U-shaped” dose-response relationship between serum HDL-C and the risk of overall all-cause mortality (37). Together with our results, we speculate that optimal circulating HDL-C level might decrease the risk for T2DM in aging subjects.

The close relationship between T2DM and abnormal lipid metabolism, and the role of lipid metabolism-related gene variants in the relationship between serum lipid and diabetes are worthy of exploration. Pérez-Martínez et al. reported that the insulin sensitivity of T2DM patients carrying *SCARB1* rs4238001 GA genotype is significantly increased as compared with those carrying GG after ingestion of monounsaturated fatty acids (23), suggesting the impact of the *SCARB1* genetic variants on modifying the susceptibility of T2DM patients to lipid metabolism. In the current study, we found that the genotype frequency of *SCARB1* rs5888 was different between the T2DM and the control subjects, as demonstrated by higher frequency of heterozygotes (GA) genotype in the T2DM patients than the control ones. The association of *SCARB1* rs5888 genetic polymorphism with the risk of T2DM was found in the dominant model, which was demonstrated by a decreased risk for T2DM in subjects with the major genotype (GG) as compared with the minor allele carrier (GA, AA) (OR = 0.636, 95% CI: 0.421–0.962). Studies reported that genetic variation of the *SCARB1* gene affected *SCARB1* gene expression, resulting in the change of circulating lipids profile, especially the level of HDL (38, 39). Smalinskiene et al. observed that male subjects with the *SCARB1* rs5888 CT genotype had significantly lower serum TG levels than the subjects with CC genotype (*P* < 0.05) (40). In our study, we did not observe the relationship between plasma TG and *SCARB1* rs5888 genetic variation. However, we found that the plasma HDL-C level of T2DM subjects carrying major genotype (GG) was significantly lower than that in control subjects. These results indicated that the major genotype (GG) of *SCARB1* rs5888 may play a protective role in T2DM through affecting plasma HDL-C level.

Significant relationship between the *CD36* rs3211938 genotype as well as the minor alleles of *CD36* SNPs (rs1984112 “G,” rs1527479 “C,” rs3211938 “G”) with clinical profiles in T2DM patients has been reported in previous study (20). The impact of *CD36* SNPs (rs1761667 “A,” rs3211938 “G,” rs1984112 “G”) on increasing body weight, BMI and the risk of T2DM was also demonstrated (41). Zhang et al.’s study observed that overweight or obesity patients carrying *CD36* rs7755 mutant allele showed increased risk for T2DM by 114% (OR = 2.14, 95% CI: 1.33–3.46; *P* = 0.007) (42). Study have found that *CD36* genetic variants are associated with serum TG and HDL-C levels in Chinese population. Subjects with rs3211848 minor genotype (AA) had higher TG level than those with major genotype (GG), and subjects with rs1054516 minor allele carrier (CC, CT) had lower HDL-C level than those with major genotype (TT) (43). In our study, we did not find the relationship between *CD36* rs1054516 genotype and HDL-C in T2DM patients. While the T2DM subjects carrying the minor genotype (CC) of *CD36* rs1054516 showed increased TC level than the subjects carrying the major genotype (TT). In contrast, in control group, increased plasma HDL-C level was observed in the subjects with the minor genotype (GG) of *CD36* rs1049673 as compared with the subjects carrying the major genotype (CC). Moreover, the serum HDL-C level of T2DM patients with *CD36* rs1049673 (CG, GG), *CD36* rs1054516 (CT), *CD36* rs2151916 (TT, CT) genotypes was lower than the control subjects. These results indicated that *CD36* genetic variants mainly affect the circulating lipid profile through the regulation of TC and HDL-C, and the T2DM subjects with *CD36* rs1049673, rs1054516, and rs2151916 genetic variants are prone to have dyslipidemia.

The study reported that fasting could induce the hepatic MFSD2A mRNA expression in a β-adrenergic signaling-dependent manner in experimental animals (44). Additionally, *MFSD2A* gene knockout mice showed slimmer bodies, increased energy expenditure, and reduced serum TG level (24), suggesting the involvement of *MFSD2A* in lipid metabolism and energy metabolism expenditure. In our study, we consistently observed the association of *MFSD2A* genetic polymorphism with lipids. We found that the T2DM subjects with *MFSD2A* rs12083239 (CG), *MFSD2A* rs4233508 (TT), and *MFSD2A* rs12072037 (AA) displayed increased plasma TG level in comparison with control subjects. Besides, the T2DM subjects with *MFSD2A* rs12083239 (GG, CG), *MFSD2A* rs4233508 (TT), and *MFSD2A* rs12072037 (CA, AA) genotypes showed lower HDL-C level than control subjects. These results indicated that the *MFSD2A* genetic variants mainly affect circulating lipids through regulating the TG and HDL-C levels, and T2DM subjects with *MFSD2A* rs12083239, rs4233508, and rs12072037 genetic variants are prone to have abnormal circulating TG and HDL-C levels.

In this study, we found that subjects with minor allele genotypes (GA, GG) of the *SCARB1* gene were associated with an increased risk of T2DM, which demonstrated that, for healthy carriers of this gene, regularly monitor of circulating lipids (especially the HDL-C level) and blood glucose levels might be benefit to reduce the risk of T2DM. Therefore, for patients with the SNPs mentioned above, more attention should be paid to lipid management to reduce the risk of dyslipidemia-based complications. This will provide a scientific basis for clinicians to efficiently prevent and treat T2DM and dyslipidemia according to individual’s genetic background.

There are several potential limitations in this study. Firstly, the study was conducted in Chinese population, the difference in lifestyle, living conditions and diet pattern makes the extrapolation of our conclusion to other population should be with caution. Secondly, environmental and genetic factors are regulators of an individual’s lipid profile, the interaction between environment and genetic factors were not measured in our study, and this should be taken into consideration during decomposing the relationship between genetic factors, serum lipids and T2DM in the future. Thirdly, the treatment and management of the T2DM subjects were another potential confounding factor, which should be considered in future large-scale cohort studies. Finally, the relatively small sample size is another limitation of the study, and population-based large scale cohort studies are needed to verify the accurate relationship between lipid metabolism-related gene polymorphism, lipids and T2DM in aging subjects in the future.

## Conclusion

In summary, our data implicated the association of lipid metabolism-related gene genetic variation, serum lipid level with T2DM in aging subjects. Genetic variants of *SCARB1* are associated with an increased risk of T2DM. Plasma TC, HDL-C and TG levels were related to *CD36*, *SCARB1* and *MFSD2A* genetic polymorphism in T2DM subjects. Large-scale population-based cohort studies are needed to reveal the biological interactional mechanism between lipid metabolism-related gene polymorphism, serum lipid level and T2DM in aging subjects.

## Data availability statement

The original contributions presented in this study are included in the article/Supplementary material, further inquiries can be directed to the corresponding author/s.

## Ethics statement

The studies involving human participants were reviewed and approved by the Committee on Medical Ethics of Capital Medical University. The patients/participants provided their written informed consent to participate in this study.

## Author contributions

LY designed the study. XW, JX, XM, YG, and HY conducted the investigation and collected the blood sample. XW performed the data statistical analysis. XW, LY, and SZ drafted the manuscript. All authors reviewed the manuscript, read, and approved the final manuscript.
